# Children with disorders of gut–brain interaction in primary care versus hospital care: A comparison of characteristics

**DOI:** 10.1002/jpn3.70129

**Published:** 2025-06-29

**Authors:** Ilse N. Ganzevoort, Marjolein Y. Berger, Arine M. Vlieger, Marc A. Benninga, Michiel R. De Boer, Gea A. Holtman

**Affiliations:** ^1^ Department of Primary and Long‐term Care, University Medical Center Groningen University of Groningen Groningen the Netherlands; ^2^ Department of Paediatrics St Antonius Hospital Nieuwegein the Netherlands; ^3^ Department of Paediatrics, Emma Children's Hospital Amsterdam University Medical Center Amsterdam the Netherlands

**Keywords:** functional abdominal pain, healthcare settings, irritable bowel syndrome, paediatric gastroenterology

## Abstract

**Objectives:**

To compare characteristics of children with functional abdominal pain (FAP) or irritable bowel syndrome (IBS) between primary and hospital care.

**Methods:**

This study makes a cross‐sectional comparison of baseline data from two randomised controlled trials (RCTs): one in Dutch primary care and one in Dutch hospital care, including secondary and tertiary care. This study included children aged 8–17 years old with FAP or IBS fulfilling the Rome III or IV criteria. Outcome measures were age, gender, Rome criteria diagnosis, duration of abdominal pain symptoms, pain intensity and frequency scores, school absenteeism, pain beliefs, somatisation score, anxiety and depression scores, and health‐related quality of life scores.

**Results:**

A total of 367 children were compared (110 in primary care, 257 in hospital care). Children seen in primary care were younger (9.8 years, 95% confidence interval [CI]: 9.4–10.7 vs. 13.6 years, 95% CI: 12.9–14.1), had a lower abdominal pain intensity score (12.0, 95% CI: 9.0–13.0 vs. 15.0, 95% CI: 15.0–16.0), frequency score (10.5, 95% CI: 9.0–13.5 vs. 16.0, 95% CI: 14.0–17.0) and somatisation score (15.0, 95% CI: 12.0–17.0 vs. 22.0, 95% CI: 20.0–25.0) compared to hospital care. A lower proportion of children had missed school because of their abdominal pain (57.3%, 95% CI: 48.2–66.4 vs. 75.9%, 95% CI: 70.4–80.9). Other characteristics were similar between groups.

**Conclusions:**

Children in primary care may differ from those in hospital care in terms of age, pain, somatisation, and school absenteeism, suggesting potential treatment response differences. Therefore, research in primary care is needed to guide evidence‐based treatment and appropriate referral decisions for general practitioners.

**Clinical Trial Registration:**

Primary care study: ClinicalTrials.gov NCT05636358. Hospital care study: Dutch Trial Register NL2597.

## Abstract (highlights)

**What is Known:**

- Available data on clinical and psychosocial differences between children with functional abdominal pain (FAP) or irritable bowel syndrome (IBS) in primary and hospital care are limited.
- Psychological treatments for FAP or IBS are studied in hospital care, but primary care studies are lacking, and generalisability remains unclear.

**What is New:**

- Children in primary care may be younger, have lower pain and somatisation scores, and experience less school absenteeism than children in hospital care.
- Evidence‐based treatments proven effective in hospital care may not show the same impact in primary care, where milder symptoms suggest a need for earlier interventions to prevent worsening of symptoms and reduce referrals.

## Graphical abstract

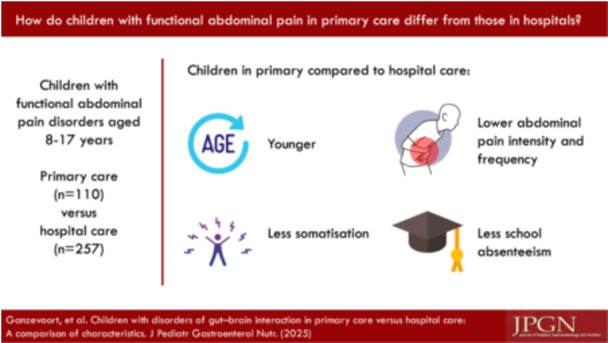

## 1 INTRODUCTION

Functional abdominal pain (FAP) and irritable bowel syndrome (IBS) are common disorders of gut–brain interaction (DGBI) in children, with a global prevalence of 13.5%.^1^ Children with DGBI are at increased risk for anxiety, depression and school absenteeism, and may experience reduced quality of life alongside increased healthcare costs.^2^, ^3^, ^4^, ^5^ In general, most patients presenting in primary care have earlier or milder symptoms compared to those seen in hospital care.^6^ Due to differences in population characteristics between children in primary and hospital care, treatments may affect pain and impact daily life differently across settings. This raises concern about the generalisability of hospital‐based treatments, such as hypnotherapy and cognitive behaviour therapy to primary care,^7^, ^8^ where their effectiveness remains unknown.

Several studies compared characteristics of children with chronic or recurrent abdominal pain across different settings, revealing contradictory findings on pain scores, symptom characteristics, functional disability, somatisation, anxiety and depression.^9^, ^10^, ^11^, ^12^ Notably, all of these studies were performed in the United States (US), where there is a lower threshold for hospital care access compared to gatekeeping healthcare systems.^13^ It remains unclear how children with FAP or IBS differ across healthcare settings within a gatekeeping healthcare system in terms of clinical and psychosocial characteristics.

Despite the fact that most children are managed in primary care, research on diagnostics and treatment within this setting is underrepresented in the literature.^14^ The aim of this study is to compare characteristics of children with FAP or IBS in primary versus hospital care. Insights into the comparative characteristics of these children may provide valuable indications for understanding potential discrepancies in treatment effects between primary care and hospital care in a gatekeeping country, where general practitioners (GPs) manage referral to hospital care.

## 2 METHODS

### 2.1 Ethics statement

The primary care randomised controlled trial (RCT) was approved by the Medical Ethics Review Committee of the University Medical Center Groningen (METc2020/237). The Medical Ethics Research Committees of all 9 participating hospitals approved the hospital care RCT. Both trials complied with the Dutch Medical Research Involving Human Subjects Act. Informed consent was obtained before completion of the baseline questionnaires: from parents for children <12 years, parents and children aged 12–15 years and children aged 16–17 years.

### 2.2 Study design and settings

This ancillary study uses baseline data from two RCTs performed in the Netherlands. These studies have large sample sizes, a similar focus, that is, hypnotherapy, and were executed in the same national gatekeeping healthcare system, which facilitated comparability. Their study protocols have been described in detail elsewhere.^15^, ^16^ The study performed in primary care studied the (cost‐)effectiveness of home‐based hypnotherapy plus care as usual versus care as usual alone in children with FAP or IBS. Children were recruited from November 2020 to September 2023 by 94 GPs from 49 practices and via media, including social media advertisements. The hospital care study took place in both secondary and tertiary care between July 2011 and June 2013. This study assessed the effect of home‐based hypnotherapy versus individual hypnotherapy given by a hypnotherapist in children with FAP or IBS.^16^, ^17^

### 2.3 Study population

This study included children aged 8–17 years with a diagnosis of FAP or IBS from two studies: one conducted in primary care and one in hospital care. In the primary care study, children aged 7–17 years participated if their GP confirmed the presence of chronic abdominal pain without an identifiable organic cause.^15^ The Rome IV criteria for FAP or IBS^18^ were subsequently assessed by the research team. In the hospital care study, children aged 8–18 years, diagnosed with FAP or IBS according to the Rome III^19^ criteria participated.^16^ FAP and IBS are characterised by recurrent abdominal pain lasting more than 2 months and for which symptoms cannot be attributed to another condition after medical evaluation. In IBS, symptoms are also accompanied by changes in stool pattern.^18^, ^19^ The Rome III and IV criteria are largely comparable, with the main difference being the frequency of abdominal pain: at least once a week in Rome III versus at least 4 days per month in Rome IV.^18^, ^19^ To ensure comparable age groups across both settings, we excluded 7‐year‐olds from the primary care study and 18‐year‐olds from the hospital care study, resulting in a final study population aged 8–17 years.

Exclusion criteria for both studies were a concomitant underlying organic gastrointestinal disease, treatment by a paediatrician (primary care study) or another healthcare professional for abdominal pain (hospital care study), previous hypnotherapy, intellectual disability and insufficient knowledge of the Dutch language.

### 2.4 Outcomes

Outcomes for this study included patient characteristics at baseline from intake meetings and questionnaires administered in both RCTs. From intake meetings, we obtained data on age, gender, proportions of FAP or IBS diagnosis based on Rome criteria, duration of complaints, and whether children had experienced school absenteeism due to abdominal complaints in the past 3 months (primary care study) or 6 months (hospital care study). These different recall periods were predefined in the original studies and could not be aligned. Questionnaires included outcomes of abdominal pain scores for intensity and frequency, pain beliefs, somatisation score, symptoms of anxiety and depression and health‐related quality of life (HR‐QoL) scores. Details of these instruments are presented in Table 1.

**Table 1:** Outcomes and instruments.

Outcome	Instrument
Abdominal pain scores	*Abdominal pain diary of 7 consecutive days* PIS: an affective facial pain scale consists of nine faces, ranging from no pain at all (score 0) to the most severe pain (score 3). The PIS is the sum score of 7 days (range 0–21). PFS: pain duration is measured in categories, ranging from no pain (score 0), 1–30 min (score 1), 31–120 min (score 2), to >120 min (score 3). The PFS is the sum score of 7 days (range 0–21).
Pain beliefs	*PBQ* ^20^ A total of 32 items consists of a pain belief statement, which ranges from not true at all (score 0) to very true (score 4). Three subscales are the sum of their items divided by the number of items (range: 0–4): pain threat (20 items), problem‐focused coping efficacy (6 items), and emotion‐focused coping efficacy (6 items). A higher score on the pain threat scale indicates stronger belief that their abdominal pain is a threat. Higher scores on both coping subscales indicate stronger beliefs in their ability to cope with pain.
Somatisation	*CSI* ^21^ Physical symptoms in the previous 2 weeks are rated on 35 items using a 5‐point Likert scale ranging from no problems (score 0) to a lot (score 4). A total score is the sum of all items, with higher scores indicating more somatic complaints (range: 0–140). Norm score for the highest decile validated in Dutch children is 19 for boys and 28 for girls.
Anxiety and depression scores	*RCADS‐25* ^22^ This questionnaire comprises 20 items of anxiety and 5 items of depression disorders. Items are rated on a 4‐point scale from 0 (never) to 3 (always). A total score is the sum of corresponding items measuring symptoms of anxiety (range: 0–60) or depression (range: 0–15). Four subscales of anxiety include generalised anxiety disorder, separation anxiety disorder, social phobia, and panic disorder. Higher scores indicate more symptoms of anxiety or depression. Norm score for the highest decile validated in Dutch children for anxiety score is at least 1 ≥10% norm score in anxiety subscales. For depression, the norm score is 5 for boys and 6 for girls.
HR‐QoL	*KIDSCREEN‐52 questionnaire* ^23^ Fifty‐two items are covered in 10 HR‐QoL dimensions: physical well‐being, psychological well‐being, moods and emotions, self‐perception, autonomy, parent relations and home life, social support and peers, school environment, social acceptance (bullying), and financial resources. Each item is rated on a 5‐point Likert scale. Rasch scores are computed for each dimension and are then converted to *T*‐value norms. Higher *T*‐values indicate a better HR‐QoL and well‐being. Norm scores for the lowest decile validated in Dutch children are: 42.23 (boys) and 39.14 (girls) for physical well‐being, 42.65 (boys) and 42.21 (girls) for psychological well‐being, 40.52 (boys) and 38.80 (girls) for moods and emotions, 43.24 (boys) and 39.38 (girls) for self‐perception, 44.88 (boys) and 43.65 (girls) for autonomy, 41.76 (boys) and 41.79 (girls) for parent relations and home life, 41.74 (boys) and 43.07 (girls) for social support and peers, 42.38 (boys) and 42.35 (girls) for school environment, 35.14 (boys) and 34.94 (girls) for social acceptance (bullying), and 39.38 (boys) and 41.43 (girls) for financial resources.

Abbreviations: CSI, children's somatisation inventory; HR‐QoL, health‐related quality of life; PBQ, pain beliefs questionnaire; PFS, pain frequency score; PIS, pain intensity score; RCADS‐25, revised anxiety and depression scale‐short version.

### 2.5 Data analysis

Characteristics of children in primary and hospital care were compared descriptively. Categorical data were presented as proportions. All variables had a non‐normal distribution; therefore, medians were reported. We calculated 95% confidence intervals (CIs) for proportions and medians using bootstrapping based on 1000 samples. Proportions of children in the worst decile based on gender‐specific norm data validated in Dutch children were determined for somatisation, anxiety and depression (highest decile) and HR‐QoL scores (lowest decile), to facilitate a comparison between groups with population norms.^21^, ^22^, ^23^ SPSS version 28.0 was used to calculate descriptive data.

## 3 RESULTS

### 3.1 Participants

Baseline data from 412 children were gathered, of which 367 were eligible for this current study: 110 children were included from the primary care study and 257 children from the hospital care study. From the primary care study, 42 children were excluded (18 had no Rome diagnosis for FAP or IBS; 24 were 7 years) and from the hospital care study, three were excluded (they were 18 years). Median age of the included population was 12.3 years (95% CI: 11.6–12.9), and 248 (67.6%) of all children were female.

### 3.2 Comparison between primary care and hospital care

**Figure 1:**
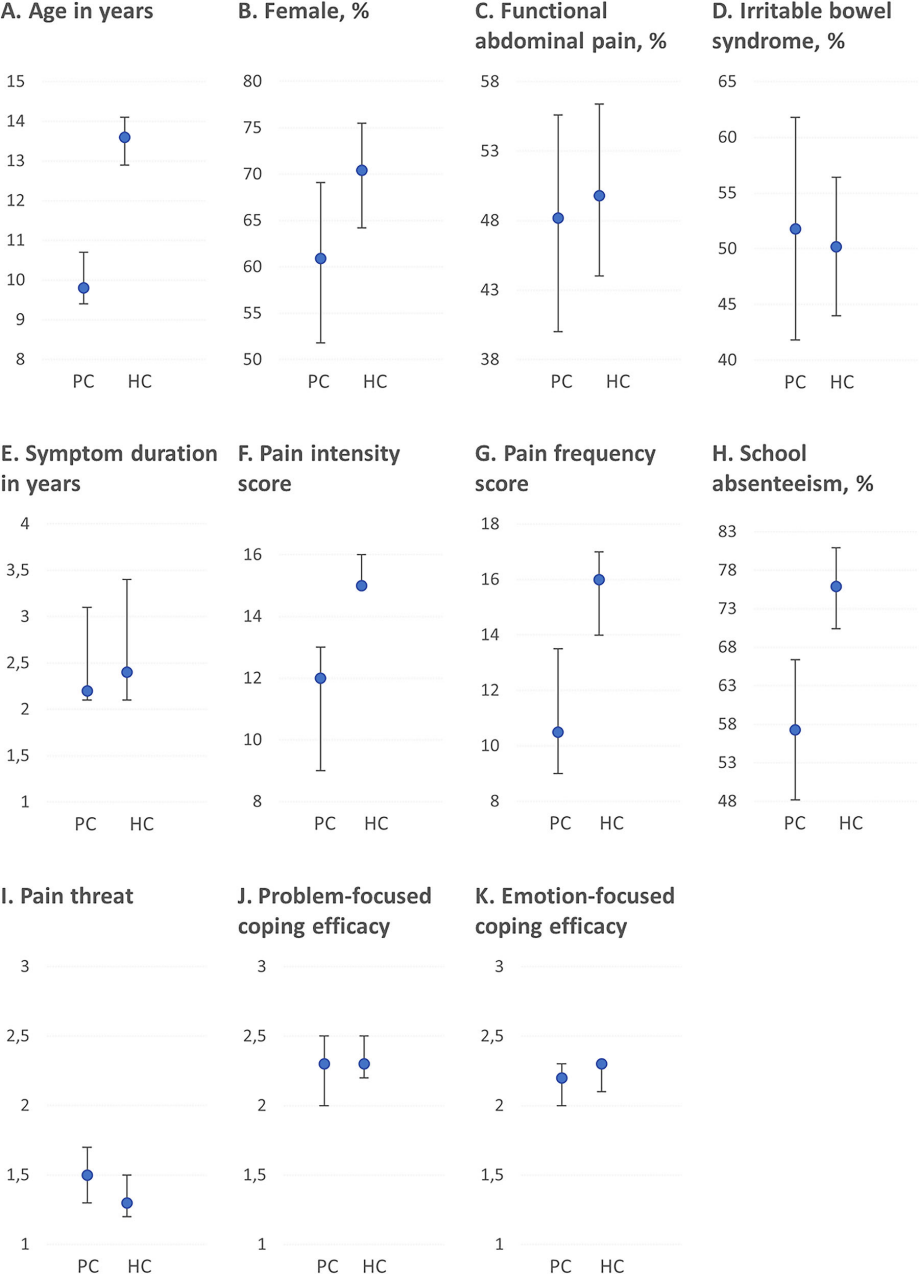
Characteristics of children in the primary care versus hospital care study. HC, hospital care; PC, primary care.

Characteristics of children in primary and hospital care are shown in Figure 1 and Table 2. Children seen in primary care had a lower median age (9.8 years, 95% CI: 9.4–10.7) compared to children seen in hospital care (13.6 years, 95% CI: 12.9–14.1). Children seen in primary care reported lower pain intensity scores (12.0, 95% CI: 9.0–13.0) and pain frequency scores (10.5, 95% CI: 9.0–13.5) compared to children seen in hospital care (15.0, 95% CI: 15.0–16.0 and 16.0, 95% CI: 14.0–17.0, respectively), but duration of symptoms was similar (2.2 years, 95% CI: 2.1–3.1 vs. 2.4 years, 95% CI: 2.1–3.4, respectively). In primary care, school absenteeism due to abdominal complaints reported over the past 3 months was lower compared to that reported in hospital care over the past 6 months (63 children, 57.3%, 95% CI: 48.2–66.4 vs. 195 children, 75.9%, 95% CI: 70.4–80.9, respectively). A table with detailed data, including medians, proportions and 95% CI, is provided in Appendix S1.

**Table 2:** Characteristics of children in the primary care versus hospital care study, including proportions of children in the worst decile based on norm data validated in Dutch children.

	*n*	Primary care study	*n*	Hospital care study
**Somatisation score**, median (95% CI)	109		255	
Total score		15.0 (12.0–17.0)		22.0 (20.0–25.0)
≥10% norm score, % (95% CI)		26.6 (18.3–34.9)		43.1 (37.3–49.8)
**Anxiety and depression score**, median (95% CI)				
Anxiety score	109	12.0 (11.0–15.0)	255	10.0 (9.0–12.0)
≥10% norm score, % (95% CI)		47.7 (38.5–56.9)		33.7 (27.5–39.6)
Depression score	109	3.0 (3.0–4.0)	255	4.0 (3.0–4.0)
≥10% norm score, % (95% CI)		21.1 (13.8–29.4)		25.1 (19.2–30.6)
**HR‐QoL**, median (95% CI)				
Physical well‐being	110	46.5 (43.7–49.5)	254	44.7 (42.53–44.7)
≤10% norm score, % (95% CI)		24.5 (17.3–32.7)		31.5 (25.63–37.0)
Psychological well‐being	110	48.9 (46.0–49.3)	255	47.1 (47.13–47.1)
≤10% norm score, % (95% CI)		26.4 (18.2–34.5)		28.6 (23.13–34.1)
Moods and Emotions	110	39.9 (38.0–43.9)	254	47.2 (45.43–49.1)
≤10% norm score, % (95% CI)		46.4 (38.2–55.5)		20.9 (16.13–26.4)
Self‐perception	110	47.1 (46.3–49.8)	254	49.8 (49.83–52.2)
≤10% norm score, % (95% CI)		13.6 (7.3–20.0)		11.0 (7.13–15.0)
Autonomy	110	50.8 (48.2–51.0)	255	50.8 (48.73–50.8)
≤10% norm score, % (95% CI)		18.2 (10.9–25.5)		21.6 (16.53–26.7)
Parent relations and home life	110	49.5 (49.4–52.1)	255	54.6 (51.83–54.6)
≤10% norm score, % (95% CI)		7.3 (2.7–12.7)		10.2 (6.73–13.7)
Social support and peers	110	52.4 (50.7–53.0)	254	48.4 (48.43–50.2)
≤10% norm score, % (95% CI)		17.3 (10.9–24.5)		19.3 (14.63–24.4)
School environment	110	49.7 (47.5–52.1)	251	50.4 (50.43–52.2)
≤10% norm score, % (95% CI)		20.0 (11.8–28.2)		12.0 (8.43–15.9)
Social acceptance (bullying)	110	48.1 (44.8–50.6)	252	58.9 (58.93–58.9)^a^
≤10% norm score, % (95% CI)		21.8 (14.5–30.0)		6.7 (4.03–9.9)
Financial resources	110	59.3 (55.4–62.9)	255	62.9 (56.33–62.9)^a^
≤10% norm score, % (95% CI)		3.6 (0.9–8.2)		7.5 (4.33–11.0)

Abbreviations: CI, confidence interval; HR‐QoL, health‐related quality of life.

^a^ ≥50% of children had a score that equals the median.

Somatisation score was lower in primary care (15.0, 95% CI: 12.0–17.0) compared to hospital care (22.0, 95% CI: 20.0–25.0). Additionally, a smaller proportion of children seen in primary care had a somatisation score in the highest decile (26.6%, 95% CI: 18.3–34.9) compared to children seen in hospital care (43.1%, 95% CI: 37.3–49.8). Anxiety scores were similar between settings, but 47.7% (95% CI: 38.5–56.9) of children seen in primary care had a score in the highest decile of norm scores for one or more of the anxiety scales, compared to 33.7% (95% CI: 27.5–39.6) of children seen in hospital care. HR‐QoL scores were similar except for the subscales ‘Moods and Emotions’ and ‘Social acceptance,’ which were lower in primary care compared to hospital care. On these subscales, more children in primary care had a score in the lowest decile compared to children in hospital care. All other outcomes were similar between settings.

### 3.3 Subgroups based on age

Characteristics of children with FAP or IBS in both settings per age group (<12 and ≥12 years) are shown in Appendix S2. Similar findings were visible for all outcomes. One exception is somatisation score, which showed smaller differences between settings in the subgroups based on age. Somatisation score in children aged below 12 years was 13.0 (95% CI: 11.0–15.5) in primary care, and 17.0 (95% CI: 14.0–19.0) in hospital care. Children aged 12 years and older had a somatisation score of 24.0 (95% CI: 16.0–30.0) in primary care versus 27.0 (95% CI: 24.0–30.0) in hospital care.

## 4 DISCUSSION

The present study found both similarities and differences in children with FAP or IBS between primary and hospital care. Although confidence intervals were wide, the results suggest that children seen in primary care were younger, had lower abdominal pain intensity and frequency scores with similar duration of symptoms, less school absenteeism, a lower somatisation score and lower HR‐QoL regarding ‘Moods and Emotions’ and ‘Social acceptance’ compared to children in hospital care. Although the median of the anxiety score was similar between settings, more children in primary care had a score in the highest decile of norm scores compared to those in hospital care.

Our results show agreement with prior studies from non‐gatekeeping countries regarding age, anxiety and depression, but they differ for symptoms, pain and somatisation in children with FAP or IBS. In line with earlier studies, children included in the primary care study were younger than those in the hospital care study.^10^, ^24^, ^25^ Children with recurrent abdominal pain at 12 years, have a two‐ to threefold increased risk for any DGBI at 16 years,^25^ older age has been found to be a predictor for referral,^10^ and older children with abdominal pain visit more healthcare services than younger children.^24^ In the primary care study, only a small proportion of children were aged 12 years or older. A prospective cohort study in primary care included a similar small proportion of children aged 10 years or older, suggesting that most children with abdominal complaints present in primary care when they are young.^26^ Therefore, age may cause potential discrepancies in psychosocial treatment effects between settings, as younger children might either respond less positively due to difficulties in understanding, or more positively due to their enhanced creativity and imagination.^27^

Our results are consistent with previous literature indicating no clear differences between settings for anxiety and depression,^9^, ^12^ while also showing higher scores in both settings compared to healthy peers.^5^, ^28^, ^29^ Although we must interpret these data with caution, we observed a higher proportion of children with an anxiety score in the worst decile in primary care compared to hospital care. This unexpected result does not seem to be influenced by young age,^30^ but might be explained by the long‐term impact of the COVID‐19 outbreak on the children in our primary care study. The primary care study was conducted after the COVID‐19 pandemic, whereas the hospital care study took place one decade earlier. It has been known that COVID‐19 led to higher anxiety and depression rates and lower quality of life in children in the general population, which seems to persist even after lockdowns.^31^ The pandemic's disruptions, such as school closures and reduced social contact, could have contributed to elevated anxiety levels in children in the primary care study.

In agreement with previous literature, children with FAP or IBS had higher school absenteeism rates and lower quality of life compared to healthy peers.^32^ The higher school absenteeism in hospital care should be interpreted with caution due to a longer recall period, but it may also reflect a greater impact on daily life and be a reason for referral. This is the first study to examine school absenteeism and quality of life across settings. Previous studies measured interference with activities because of abdominal pain and functional disability, but found no differences between children in different care settings.^11^, ^12^ However, two other studies found less functional disability in primary care compared to hospital care.^9^, ^10^

Compared to previous literature, this study found two unexpected results. First, we found no clear difference in symptom duration across settings, whereas a study performed in the US found that referral was best predicted by child‐reported abdominal pain duration.^10^ Many children in the Netherlands have chronic abdominal pain symptoms, with 50% of children still reporting abdominal pain having an impact on daily activities 1 year after first presentation.^26^, ^33^ Additionally, the absence of a difference in the present study could be explained by the recruitment method of the primary care study, which included children recruited through advertisements and social media due to challenges in obtaining participants via GPs.^15^ This self‐enrolment suggests that children had been experiencing abdominal pain for a longer period before seeking help, rather than presenting with newly onset symptoms.

Second, we found lower somatisation scores for children in primary care compared to hospital care, whereas previous literature did not find differences between settings.^9^, ^10^, ^12^ This disagreement could be explained by age and who reported the somatisation score. While it is known that children with FAP or IBS have higher somatisation scores than healthy peers,^34^ the present study additionally shows potential higher scores in older than in younger children, taking low precision into account. In our study, children over 12 years completed the questionnaires themselves, but for younger children, it is unclear whether responses were child‐ or parent‐reported, or both because they were stimulated to complete questionnaires together. One study found no group differences for child‐reported somatisation, while parent‐reported somatisation was worse for the hospital group.^10^ Overall, there is good agreement between child‐and parent report for somatisation and quality of life at a group level, but individual differences exist.^35^, ^36^ Therefore, parental influence on reported outcomes may play a role and have influenced our study results.

A strength of this study is that we were able to compare a large set of baseline characteristics from two extensive RCTs. Both studies used the same outcome measures, which enabled direct comparison. Additionally, most outcomes were assessed using validated questionnaires, which allowed for the identification of children scoring within the worst tenth percentile validated in a Dutch sample. This enabled a direct comparison between children in primary and hospital care settings and the general population, enhancing the interpretability of our findings. However, this study selected children aged 8–17 years with a diagnosis of FAP or IBS according to Rome criteria. Therefore, a potential limitation could be that the treatment might be applied more broadly in clinical practice, potentially extending to children outside the study's specific age range and those without a formal Rome diagnosis, as these criteria are not commonly applied in routine clinical practice.^37^ Another limitation of this study is that it was not powered for statistical testing, because the RCTs had another primary aim. We did show 95% CI to indicate the level of precision in our findings.^38^ A final limitation is that these studies were performed over 10 years apart, including the COVID‐19 pandemic. Recruitment for the primary care study proved challenging, partly due to the pandemic, leading to the addition of media‐based recruitment. Timing between the studies likely had an impact on consulting behaviour, healthcare organisation^39^ and psychosocial factors,^40^ which could have influenced study results.

## 5 CONCLUSIONS

This study highlights the importance of performing research not only in hospital care but also in primary care, because factors such as age, pain, somatisation and school absenteeism might differ across settings. Children in primary care are younger than children in hospital care and may respond differently to psychological treatments. Additionally, our data show that they have lower baseline pain scores and less school absenteeism, implying that there might be less to gain from treatment compared to referred children. Early intervention in primary care may help prevent symptom worsening, potentially reducing the need for hospital referrals. Concluding, GPs need evidence‐based treatment options assessed in primary care to help in deciding between a wait‐and‐see policy, offering treatments or referring to hospital care. Therefore, research in children with FAP or IBS in primary care is needed, and this study could help explain potential differences found in treatment effects between settings.

## CONFLICT OF INTEREST STATEMENT

The authors declare no conflicts of interest.

## Supporting information

Supplemental material.
