# Supplementary material for: Children with disorders of gut–brain interaction in primary care versus hospital care: A comparison of characteristics
Source: J Pediatr Gastroenterol Nutr. 2025 Jun 29;81(3):530–9. doi: 10.1002/jpn3.70129 (PMC12408964; doi:10.1002/jpn3.70129)
Supplement: Supplementary file 1 — Supplemental material. [file JPN3-81-530-s001.docx]

**appendiCES**

**Appendix 1. Detailed characteristics of children in the primary care versus hospital care study**

|  | **n** | **Primary care study** | **n** | **Hospital care study** |
| --- | --- | --- | --- | --- |
| **Age**, years, median (95% CI) | 110 | 9.8 (9.4-10.7) | 257 | 13.6 (12.9-14.1) |
| **Female**, n (%; 95% CI) | 110 | 67 (60.9; 51.8-69.1) | 257 | 181 (70.4; 64.2-75.5) |
| **Rome diagnosis**, n (%) | 110 |  | 257 |  |
| FAP |  | 53 (48.2; 40.0-55.6) |  | 128 (49.8; 44.0-55.6) |
| IBS |  | 57 (51.8; 41.8-61.8) |  | 129 (50.2; 44.0-56.4) |
| **Symptom duration**, years, median (95% CI) | 104 | 2.2 (2.1-3.1) | 254 | 2.4 (2.1-3.4) |
| **Abdominal pain score**, median (95% CI) |  |  |  |  |
| PIS | 85 | 12.0 (9.0-13.0) | 257 | 15.0 (15.0-16.0) |
| PFS | 84 | 10.5 (9.0-13.5) | 257 | 16.0 (14.0-17.0) |
| **School absenteeism**, n (%) | 110 | 63 (57.3; 48.2-66.4) | 257 | 195 (75.9; 70.4-80.9) |
| **Pain beliefs score**, median (95% CI) |  |  |  |  |
| Pain threat | 109 | 2.2 (2.0-2.3) | 250 | 2.3 (2.1-2.3) |
| Problem-focused coping efficacy | 109 | 1.5 (1.3-1.7) | 255 | 1.3 (1.2-1.5) |
| Emotion-focused coping efficacy | 109 | 2.3 (2.0-2.5) | 255 | 2.3 (2.2-2.5) |

CI: confidence interval; FAP: functional abdominal pain; IBS: irritable bowel syndrome; PIS: pain intensity score; PFS: pain frequency score.

**Appendix 2. Characteristics of subgroups based on age**

|  | **< 12-year-old children** | | | | **≥ 12-year-old children** | | | |  |
| --- | --- | --- | --- | --- | --- | --- | --- | --- | --- |
|  | **n** | **Primary care study** | **n** | **Hospital care study** | **n** | **Primary care study** | **n** | **Hospital care study** | |
| **Age in years**, median (95% CI) | 83 | 9.3 (9.0-9.6) | 96 | 10.5 (10.0-10.8) | 27 | 14.3 (13.3-14.6) | 161 | 15.2 (14.9-15.5) | |
| **Female**, n (%; 95% CI) | 83 | 50 (60.2; 48.9-70.8) | 96 | 59 (61.5; 51.1-71.0) | 27 | 17 (63.0; 43.5-81.0) | 161 | 122 (75.8; 68.9-82.1) | |
| **Rome diagnosis**, n (%; 95% CI) | 83 |  | 96 |  | 27 |  | 161 |  | |
| FAP |  | 44 (53.0; 43.2-64.3) |  | 48 (50.0; 40.4-59.6) |  | 9 (33.3; 14.8-53.6) |  | 80 (49.7; 42.3-57.7) | |
| IBS |  | 39 (47.0; 35.7-58.3) |  | 48 (50.0; 40.4-59.5) |  | 18 (66.7; 50.0-82.8) |  | 81 (50.3; 42.6-58.1) | |
| **Symptom duration**, years, median (95% CI) | 79 | 2.1 (1.8-2.7) | 95 | 3.0 (2.0-4.1) | 25 | 3.2 (0.7-5.4) | 159 | 2.3 (2.1-3.1) | |
| **Abdominal pain score**, median (95% CI) |  |  |  |  |  |  |  |  | |
| PIS | 66 | 13.0 (10.0-14.0) | 96 | 15.0 (14.0-16.0) | 19 | 9.0 (6.0-13.0) | 161 | 15.0 (14.0-17.0) | |
| PFS | 66 | 13.0 (9.0-15.0) | 96 | 14.0 (13.0-16.0) | 18 | 8.5 (6.0-13.0) | 161 | 16.0 (15.0-18.0) | |
| **School absenteeism**, n (%; 95% CI) | 83 | 47 (56.6; 46.0-67.0) | 96 | 63 (65.6; 56.0-74.5) | 27 | 16 (59.3; 40.0-77.3) | 161 | 132 (82.0; 76.3-87.5) | |
| **Pain beliefs score**, median (95% CI) |  |  |  |  |  |  |  |  | |
| Pain threat | 83 | 2.2 (2.1-2.5) | 95 | 2.1 (1.9-2.2) | 26 | 2.0 (1.7-2.3) | 155 | 2.4 (2.3-2.5) | |
| Problem-focused coping efficacy | 83 | 1.3 (1.0-1.5) | 95 | 1.7 (1.5-1.8) | 26 | 1.8 (1.3-2.0) | 160 | 1.2 (1.0-1.3) | |
| Emotion-focused coping efficacy | 83 | 2.2 (2.0-2.3) | 95 | 2.5 (2.2-2.7) | 26 | 2.5 (2.0-2.7) | 160 | 2.3 (2.0-2.3) | |
| **Somatisation score**, median (95% CI) | 83 |  | 95 |  |  |  |  |  | |
| Total score |  | 13.0 (11.0-15.5) |  | 17.0 (14.0-19.0) | 26 | 24.0 (16.0-30.0) | 160 | 27.0 (24.0-30.0) | |
| ≥10% norm score, % (95% CI) |  | 21.7 (12.8-30.5) |  | 28.4 (19.4-37.4) |  | 42.3 (23.1-60.9) |  | 51.9 (43.2-59.6) | |
| **Anxiety and depression score**, median (95% CI) |  |  |  |  |  |  |  |  | |
| Anxiety total score | 83 | 12.0 (10.0-15.0) | 95 | 9.0 (8.0-11.0) | 26 | 13.5 (10.0-17.0) | 160 | 10.5 (9.0-12.0) | |
| ≥10% norm score, % (95% CI) |  | 50.6 (39.8-61.5) |  | 34.7 (24.7-44.7) |  | 38.5 (20.0-57.1) |  | 33.1 (26.2-40.4) | |
| Depression total score | 83 | 3.0 (3.0-4.0) | 95 | 3.0 (3.0-4.0) | 26 | 3.0 (2.0-5.0) | 160 | 4.0 (3.0-4.0) | |
| ≥10% norm score, % (95% CI) |  | 19.3 (10.5-28.2) |  | 17.9 (10.5-25.9) |  | 26.9 (10.7-45.0) |  | 29.4 (21.5-36.4) | |
| **HR-QoL score**, median (95% CI) |  |  |  |  |  |  |  |  | |
| Physical well-being | 83 | 46.5 (43.7-52.7) | 95 | 49.6 (47.1-52.4) | 27 | 44.7 (42.5-47.1) | 159 | 42.5 (40.5-42.5) | |
| ≤10% norm score, % (95% CI) |  | 26.5 (17.3-35.9) |  | 17.9 (10.2-25.8) |  | 18.5 (5.0-34.3) |  | 39.6 (31.9-47.1) | |
| Psychological well-being | 83 | 48.9 (46.0-52.1) | 95 | 51.8 (49.3-54.5) | 27 | 47.1 (44.2-49.3) | 160 | 45.1 (43.2-47.1) | |
| ≤10% norm score, % (95% CI) |  | 27.7 (18.3-37.0) |  | 15.8 (9.1-23.5) |  | 22.2 (8.0-39.1) |  | 36.3 (28.8-44.1) | |
| Moods and Emotions | 83 | 38.0 (36.2-43.9) | 95 | 51.3 (49.1-54.0) | 27 | 43.9 (40.0-48.1) | 159 | 45.4 (42.5-47.2) | |
| ≤10% norm score, % (95% CI) |  | 50.6 (39.5-61.3) |  | 12.6 (6.6-19.4) |  | 33.3 (16.7-52.0) |  | 25.8 (19.5-32.9) | |
| Self-perception | 83 | 49.1 (46.5-52.3) | 95 | 55.4 (55.4-60.1) | 27 | 46.1 (41.8-49.8) | 159 | 47.8 (46.1-48.8) | |
| ≤10% norm score, % (95% CI) |  | 13.3 (6.3-21.2) |  | 1.1 (0.0-3.3) |  | 14.8 (3.3-29.2) |  | 17.0 (11.0-23.0) | |
| Autonomy | 83 | 51.0 (48.2-51.0) | 95 | 50.8 (48.7-54.7) | 27 | 48.7 (45.2-50.8) | 160 | 48.7 (46.9-50.8) | |
| ≤10% norm score, % (95% CI) |  | 15.7 (7.9-24.0) |  | 12.6 (6.1-20.2) |  | 25.9 (10.5-44.1) |  | 26.9 (20.3-33.5) | |
| Parent relations and home life | 83 | 49.4 (49.4-52.1) | 95 | 58.5 (54.6-58.5) | 27 | 51.8 (47.5-58.5) | 160 | 51.8 (49.5-54.6) | |
| ≤10% norm score, % (95% CI) |  | 6.0 (1.2-11.6) |  | 3.2 (0.0-6.9) |  | 11.1 (0.0-25.0) |  | 14.4 (9.3-20.1) | |
| Social support and peers | 83 | 53.0 (50.7-53.0) | 95 | 50.2 (48.4-52.4) | 27 | 46.7 (43.6-52.4) | 159 | 48.4 (46.7-50.2) | |
| ≤10% norm score, % (95% CI) |  | 15.7 (8.1-23.5) |  | 17.9 (10.0-25.5) |  | 22.2 (7.4-37.9) |  | 20.1 (14.2-26.3) | |
| School environment | 83 | 49.7 (47.5-52.1) | 94 | 55.3 (53.2-58.9) | 27 | 46.9 (43.8-52.2) | 157 | 50.4 (46.9-50.4) | |
| ≤10% norm score, % (95% CI) |  | 16.9 (8.9-25.6) |  | 5.3 (1.1-10.2) |  | 29.6 (11.6-48.0) |  | 15.9 (10.1-21.7) | |
| Social acceptance (bullying) | 83 | 44.8 (44.8-50.6) | 94 | 58.8 (58.8-58.8) | 27 | 48.1 (42.2-58.8) | 158 | 58.8 (58.8-58.8) | |
| ≤10% norm score, % (95% CI) |  | 24.1 (15.2-34.3) |  | 5.3 (1.1-10.2) |  | 14.8 (3.4-30.4) |  | 7.6 (3.8-12.1) | |
| Financial resources | 83 | 59.3 (55.4-65.0) | 95 | 62.9 (59.6-62.9) | 27 | 62.9 (49.3-62.9) | 160 | 56.3 (52.4-62.9) | |
| ≤10% norm score, % (95% CI) |  | 2.4 (0.0-6.3) |  | 10.5 (4.8-16.7) |  | 7.4 (0.0-20.0) |  | 5.6 (2.4-9.4) | |

CI: confidence interval; FAP = functional abdominal pain; IBS = irritable bowel syndrome; PIS = pain intensity score; PFS = pain frequency score; HR-QoL = health-related quality of life.
